# Supplementary material for: Differential Amino Acid Uptake and Depletion in Mono-Cultures and Co-Cultures of Streptococcus thermophilus and Lactobacillus delbrueckii subsp. bulgaricus in a Novel Semi-Synthetic Medium
Source: Microorganisms. 2022 Sep 1;10(9):1771. doi: 10.3390/microorganisms10091771 (PMC9505316; doi:10.3390/microorganisms10091771)
Supplement: Supplementary file 1 [file microorganisms-10-01771-s001.zip › microorganisms-1881764-supplementary.pdf]

Supplementary Materials

Differential Amino Acid Uptake and Depletion in Mono-Cultures and Co-Cultures of *Streptococcus thermophilus* and *Lactobacillus delbrueckii* subsp. *bulgaricus* in a Novel Semi-Synthetic Medium

Ulmer, A.; Erdemann, F.; Mueller, S.; Wildt, S.; Loesch, M.; Lund Jensen, M.; Gaspar, P.; Zeidan, A. A. and Takors, R.

Table S1. Amino acid concentrations (µM) bound in extracellular peptides and casein determined at two specific time points in cocultivation of LB.1 with proteinase-positive ST.1 in SMcas. Supernatants were hydrolysed and amino acid concentrations were determined. Then, free amino acid concentrations measured in non-hydrolysed supernatants were subtracted.

µM	Time (hour)	asp	glu	ser	his	gly	thr	Arg+ala	val	phe	ile	leu	lys
LB.1+ST.1 coculture	0	1136	3312	1185	361	520	751	1133	1112	664	803	1502	**1133**
LB.1+ST.1 coculture	4	309	1469	632	182	300	239	64	998	116	101	308	**230**

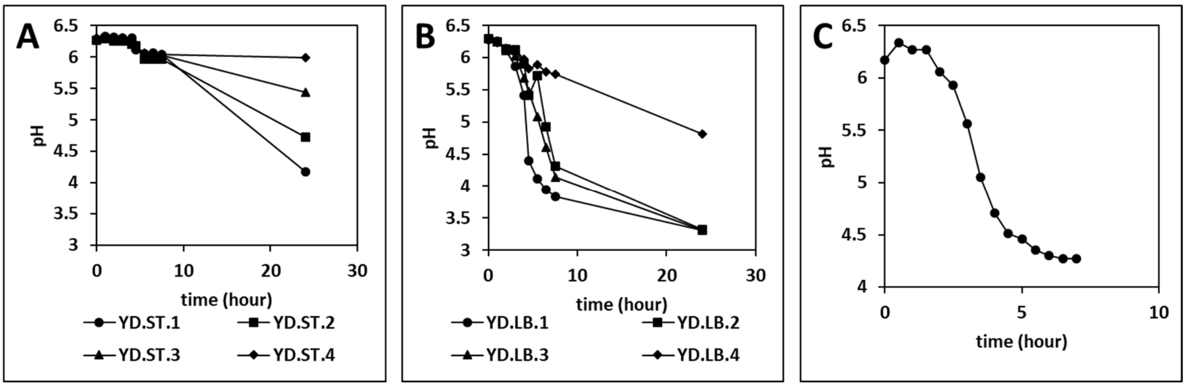

Figure S1. Acidification of SMcas medium by different strains of *S. thermophilus* (A) and *L. bulgaricus* in SMcas (B), as well as of SMaa medium by the proteinase-negative *S. thermophilus* ST.4 (C). The experimental conditions were according to method section. (unreplicated experiments)

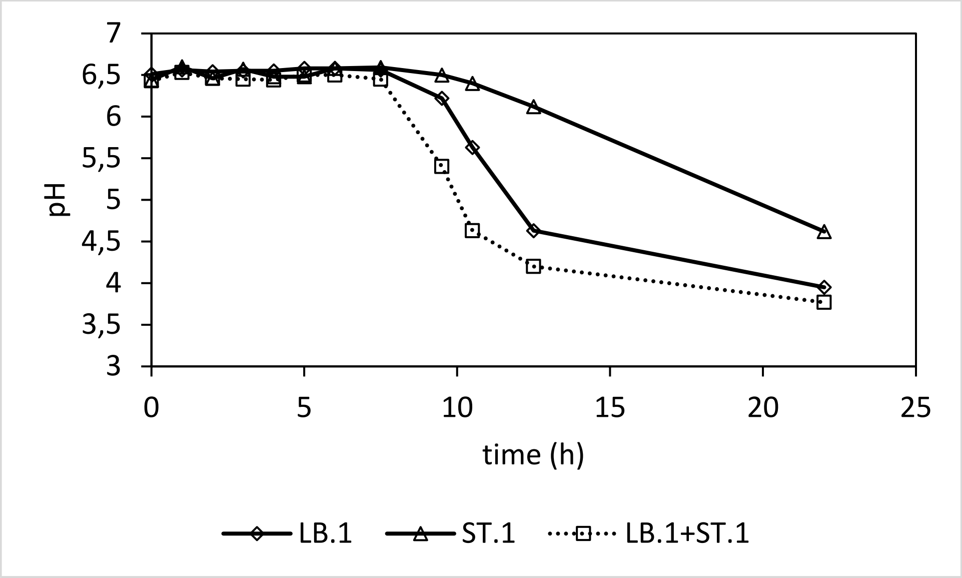

Figure S2. Acidification of SMcas. LB.1 mono-culture (rhomb), ST.1 mono-culture (triangle), or LB.1 – ST.1 co-culture (square). The co-culture was inoculated with the same biomass as the respective mono-cultures. The experimental conditions were according to method section. (unreplicated experiments).

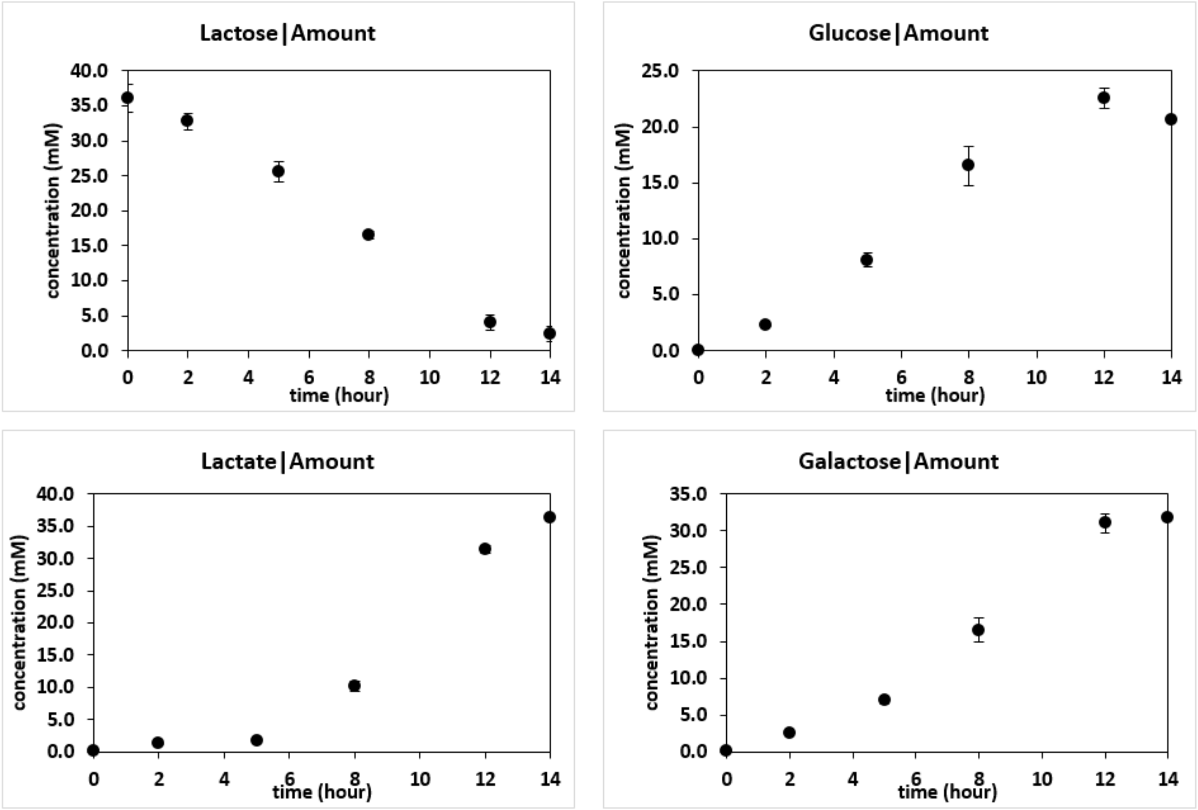

Figure S3. Extracellular metabolite concentrations along growth of proteinase-positive *S. thermophilus* ST.1 in SMcas. The experimental conditions were according to method section.

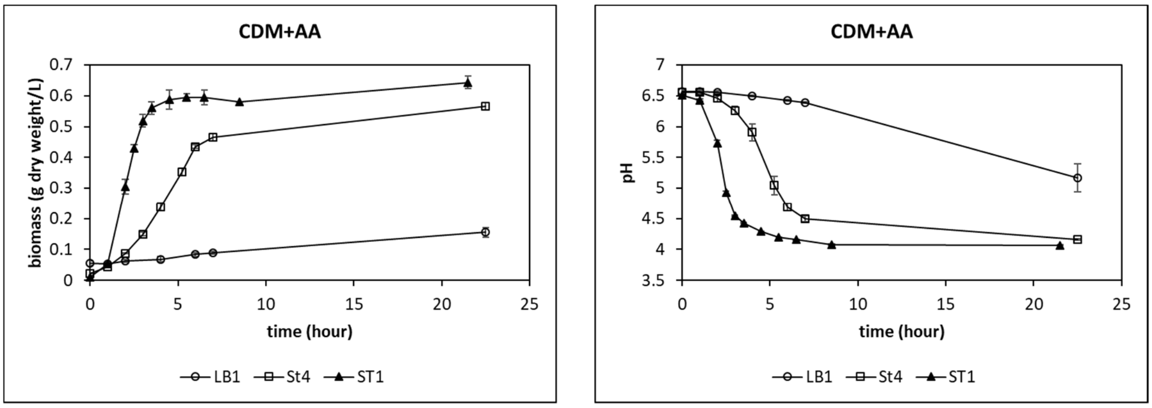

Figure S4. Acidification of SMaa and biomass profiles of LB.1 (triangle), ST.1 (square), and ST.4 (circle). The experimental conditions were according to method section.

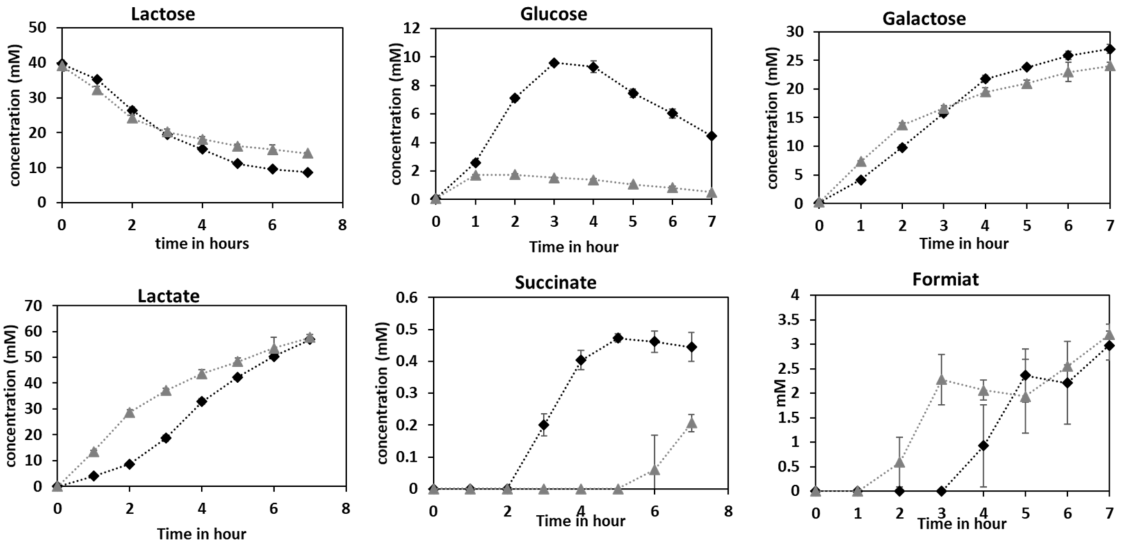

Figure S5. Profiles of extracellular metabolite concentrations in cocultivations of LB.1 with ST.1 (rhomb), and LB.1 with ST.4 (triangle) in SMcas. The experimental conditions were according to method section.

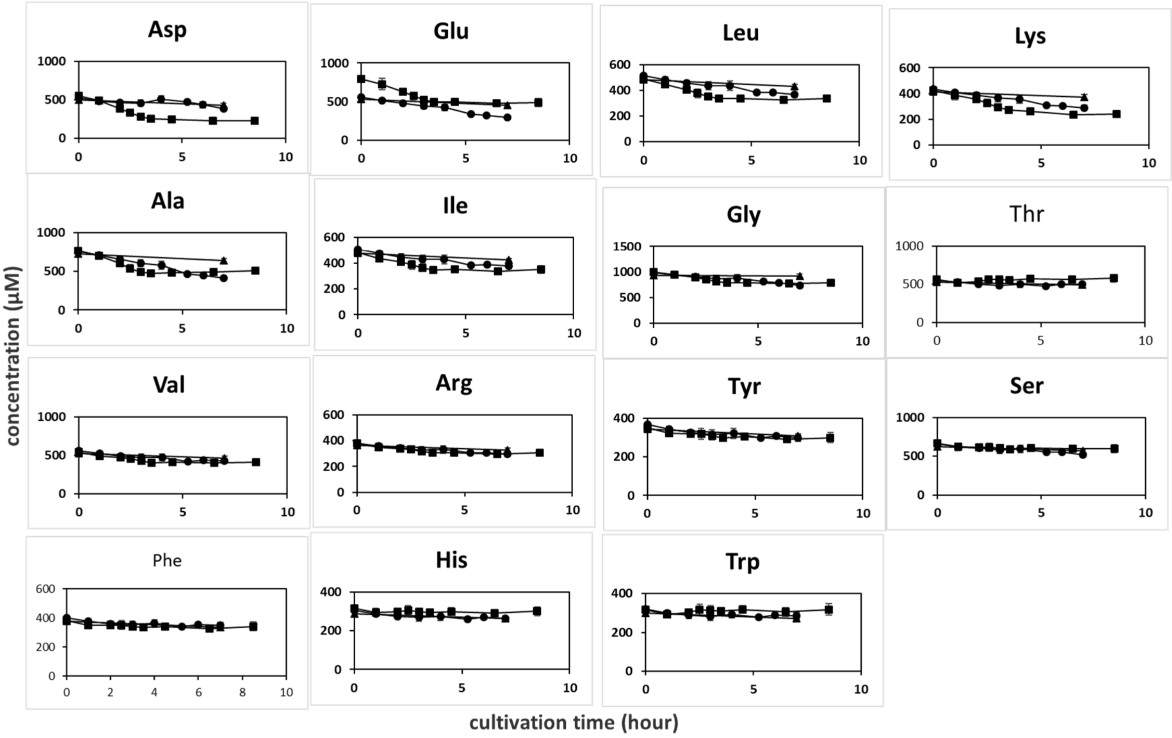

Figure S6. Profiles of amino acids along growth of LB.1 (triangle), ST.1 (square), and ST.4 (circle) in SMaa. The experimental conditions were according to method section.

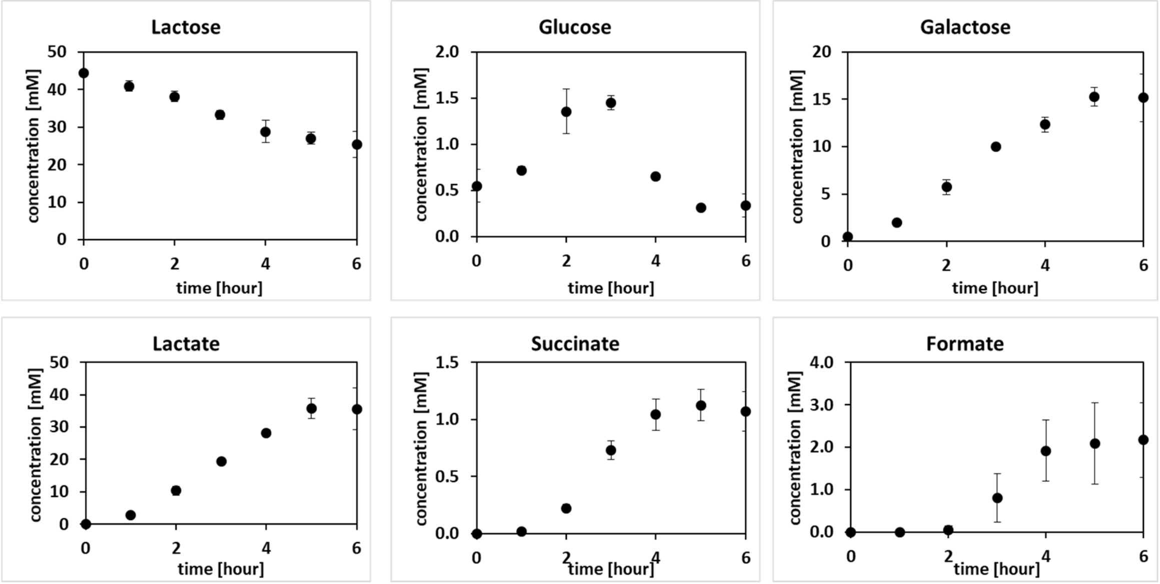

Figure S7. Profiles of extracellular metabolites along growth of *L. bulgaricus* LB.1 in SMcas. The experimental conditions were according to method section.

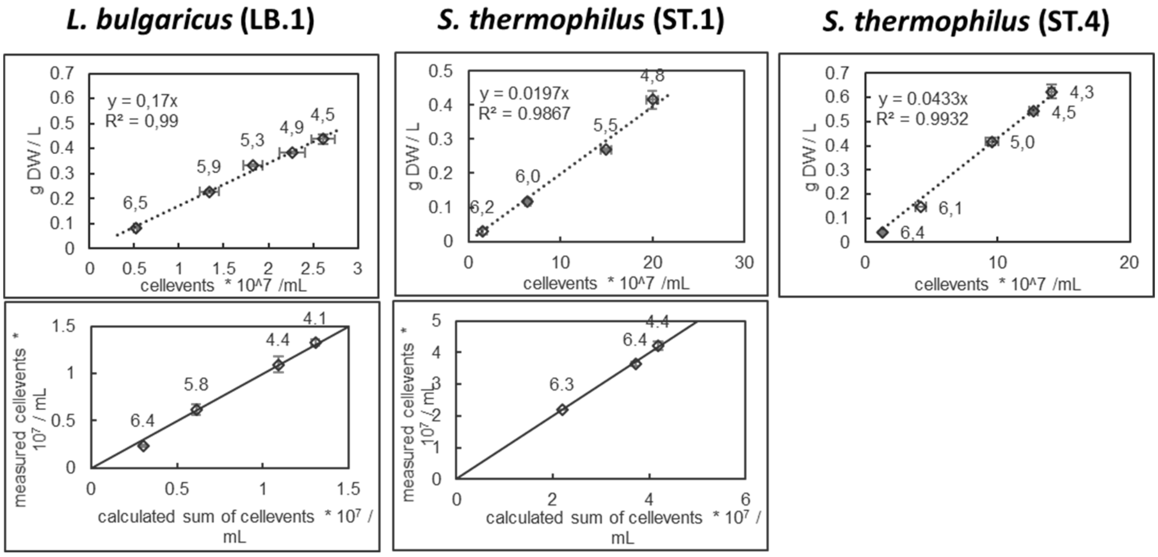

Figure S8. Top row: correlations between flow cytometry data and cell dry weight of cells grown in SMaa during acidification (data point labels indicate pH of samples). The experimental conditions were according to method section. **Bottom row**: x-axis: calculated sum of cells grown in SMaa plus cells grown in SMcas; y-axis: total cell events of mixed samples. The mean deviation between calculated and measured total cell events was 6±11 % for LB.1 and 1±1 % for ST.1. Data point labels indicate pH of samples in SMcas. Flow cytometry data and dry weight are given as means of three technical replicates (n = 3) ± standard deviations.

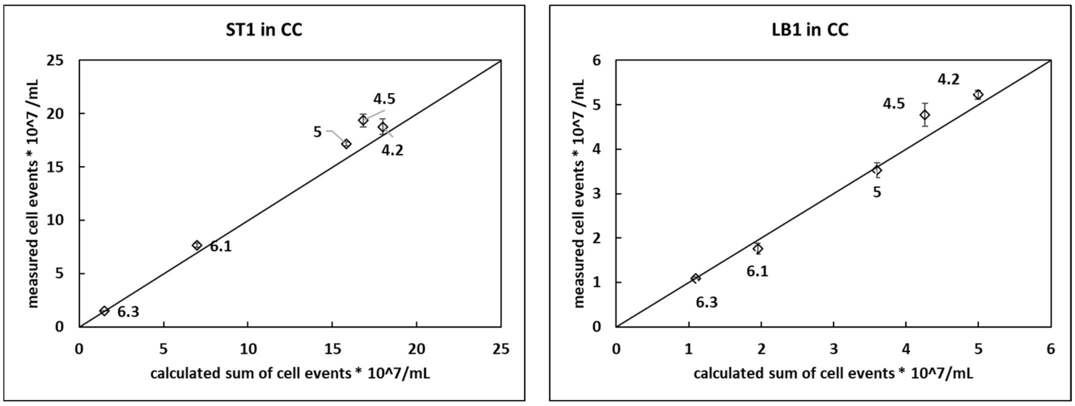

Figure S9. Enumeration of total cell events in ST.1-LB.1-co-culture mixed with known amounts of ST.1 (left) or LB.1 (right). The x-axis value indicates the calculated sum of total cell events in co-culture plus mono-culture sample. The y-axis value indicates the measured total cell events in co-culture mixed with mono-culture. The mean deviation between calculated and measured total cell events was 8±6% for ST.1 and 6±5% for LB.1. Data point labels indicate pH of co-culture sample. The variance indicates technical duplicates. Flow cytometry data are given as means of three technical replicates (n = 3) ± standard deviations.

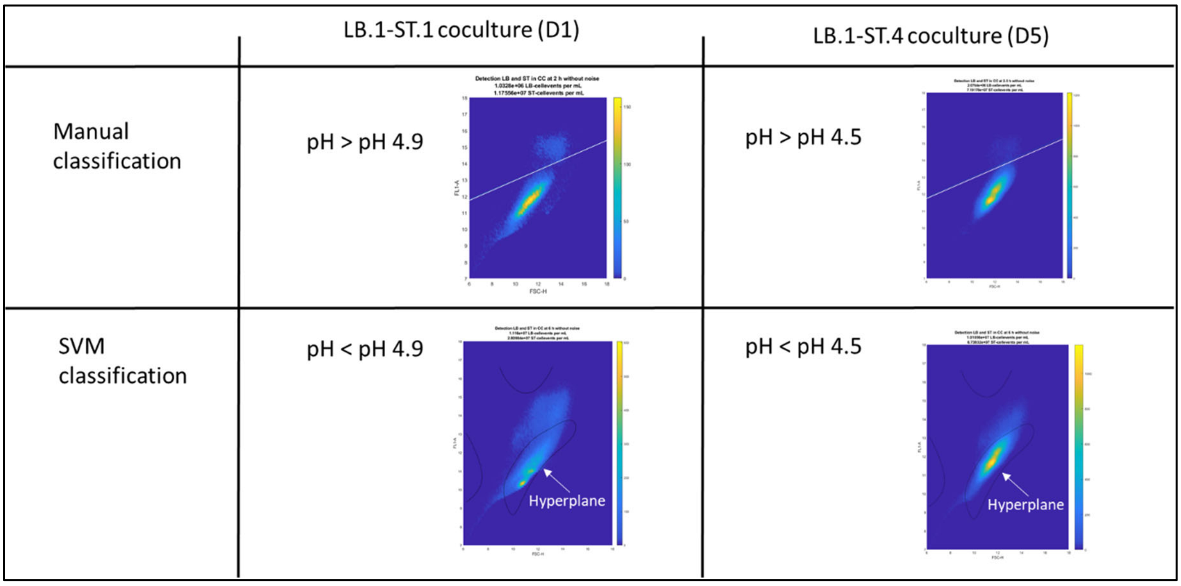

Figure S10. Exemplarily classification of flow cytometry data as *S. thermophilus* or *L. bulgaricus* (data points originating from casein were removed previously). (left) The manual classification by linear line was applied on samples with a pH ≥ 4.9 The SVM was applied on samples with a pH ≤ 4.9 for LB.1-ST.1 co-culture samples. (right) The manual classification by linear line was applied on samples with a pH ≥ 4.5. The SVM was applied on samples with a pH ≤ 4.5 for LB.1-ST.4 co-culture samples.

```
c = cvpartition(d,'KFold',10);

opts = struct('Optimizer','bayesopt','ShowPlots',true,'CVPartition',c,...
'AcquisitionFunctionName','expected-improvement-plus');

svm_LBST = fitcsvm(cdata,grp,'Standardize',true,'KernelFunction','rbf',...
'OptimizeHyperparameters','auto','HyperparameterOptimizationOptions',opts)
```

Figure S11. MATLAB code for SVM training with an optimizer for the identification of *L. bulgaricus* and *S. thermophilus* in co-culture. cdata = training data set containing 1000 events of the FL1-A and FSC-H signals of the flow cytometry data of a sample of *L. bulgaricus* and *S. thermophilus* (if the data set of the flow cytometry data of the samples is smaller it contains accordingly less events), “grp” = vector of the length of cdata containing ones and minus ones labelling the cdata (1 = *L. bulgaricus*, -1 = *S. thermophilus*).

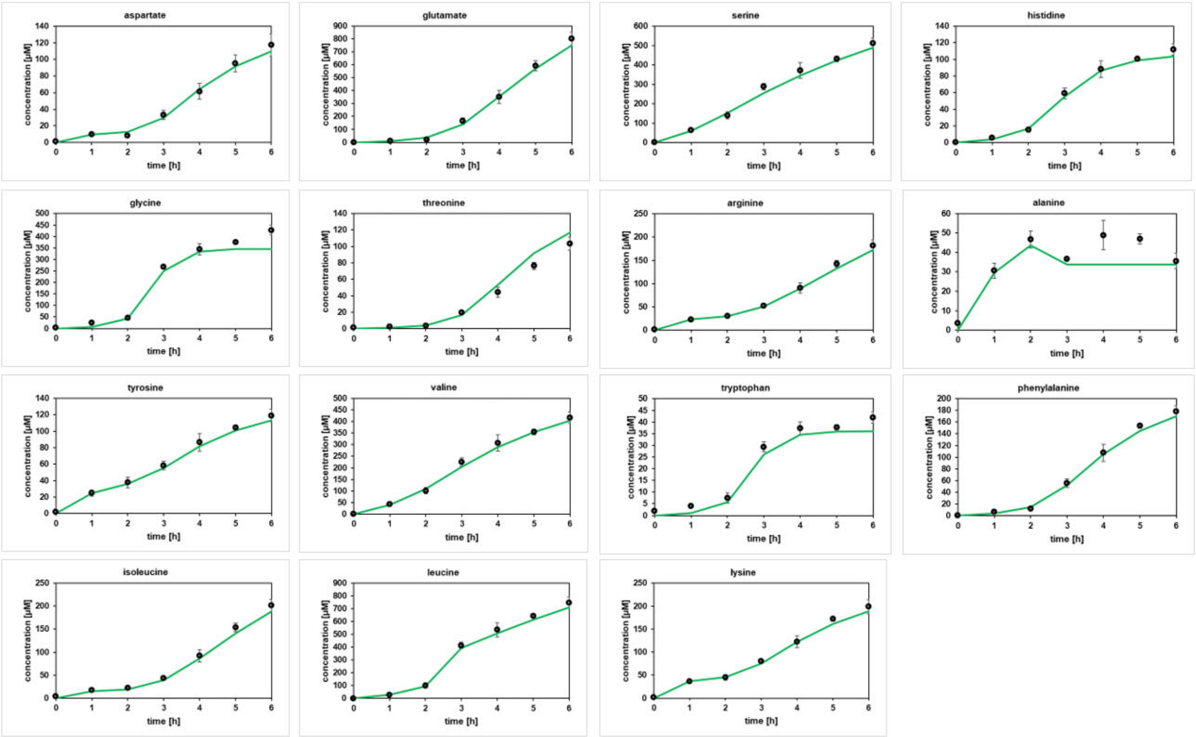

Figure S12. Patterns of amino acids concentrations along growth of *L. bulgaricus* LB.1 in SMcas (dots). Simulated amino acid concentrations based on biomass and pH-dependent Gaussian model for amino acid release (line).

General model Gauss2:

$$val(x) = a1*\exp(-((x-b1)/c1)^2) + a2*\exp(-((x-b2)/c2)^2)$$

Coefficients:

a1 = 5.946e+16
b1 = 8.41
c1 = 0.347
a2 = 88.37
b2 = 5.185
c2 = 0.7943

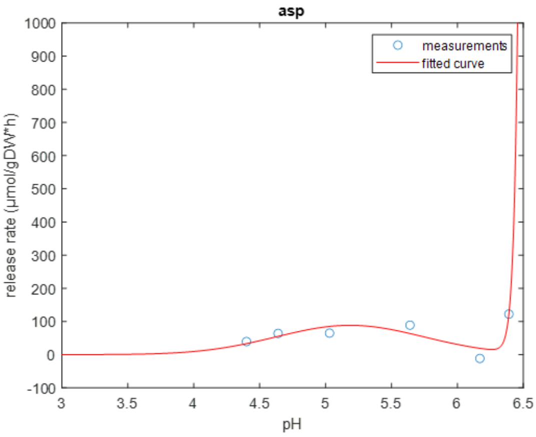

Figure S13. fitted Gauss model for aspartate.

General model Gauss1:
val(x) = a1*exp(-((x-b1)/c1)^2)
Coefficients (with 95% confidence bounds):
a1 = 520 (240.7, 799.3)
b1 = 5.076 (4.603, 5.549)
c1 = 1.048 (0.257, 1.839)

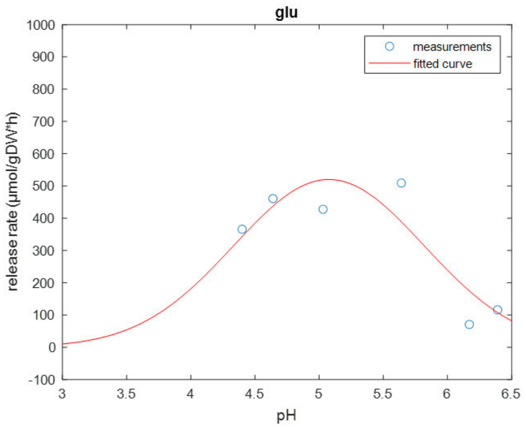

Figure S14. fitted Gauss model for glutamate.

General model Gauss1:
val(x) = a1*exp(-((x-b1)/c1)^2)
Coefficients (with 95% confidence bounds):
a1 = 5.929e+59 (-8.523e+64, 8.523e+64)
b1 = 297.1 (-3.179e+05, 3.185e+05)
c1 = 25.4 (-1.384e+04, 1.389e+04)

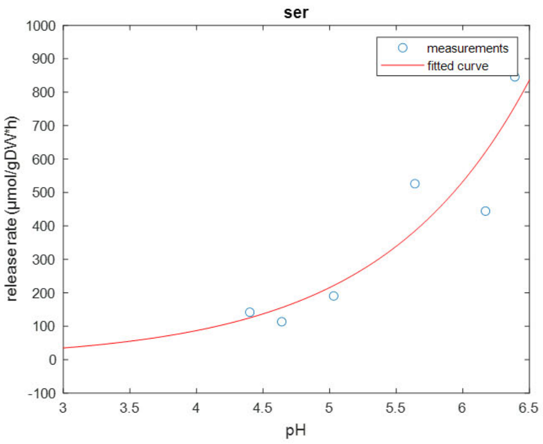

Figure S15. fitted Gauss model for serine.

General model Gauss1:
val(x) = a1*exp(-((x-b1)/c1)^2)
Coefficients (with 95% confidence bounds):
a1 = 144.9 (73.04, 216.9)
b1 = 5.626 (5.364, 5.889)
c1 = 0.738 (0.3859, 1.09)

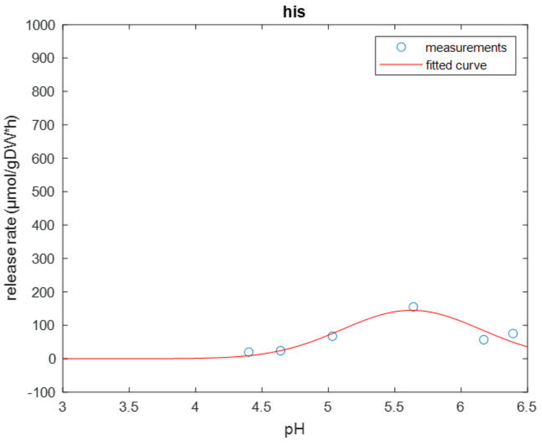

Figure S16. fitted Gauss model for histidine.

General model Gauss1:
  val(x) = a1*exp(-((x-b1)/c1)^2)
  Coefficients (with 95% confidence bounds):
    a1 =   770.4 (285.4, 1255)
    b1 =   5.618 (5.283, 5.954)
    c1 =   0.5148 (0.2046, 0.825)

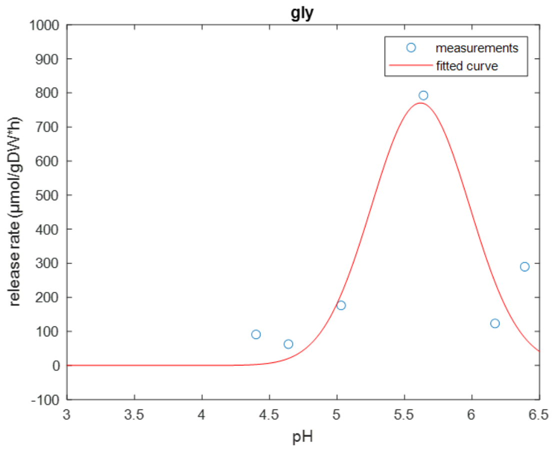

Figure S17. fitted Gauss model for glycine.

General model Gauss1:
  val(x) = a1*exp(-((x-b1)/c1)^2)
  Coefficients (with 95% confidence bounds):
    a1 =   64.48 (37.42, 91.54)
    b1 =   5.014 (4.579, 5.449)
    c1 =   1.087 (0.3788, 1.795)

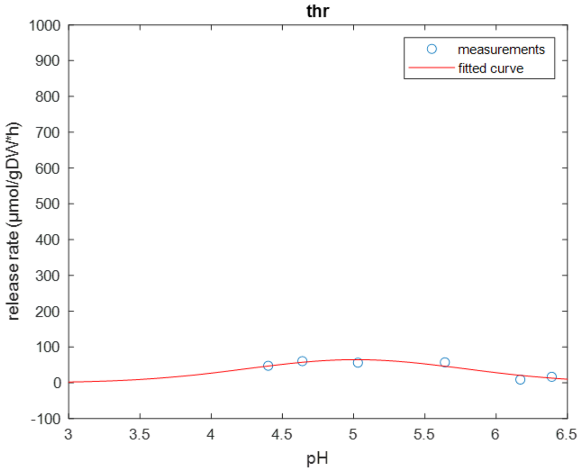

Figure S18. fitted Gauss model for threonine.

General model Gauss2:
  val(x) = a1*exp(-((x-b1)/c1)^2) + a2*exp(-((x-b2)/c2)^2)
  Coefficients:
    a1 =   2.827e+05
    b1 =   7.111
    c1 =   0.2724
    a2 =   94.37
    b2 =   5.028
    c2 =   1.325

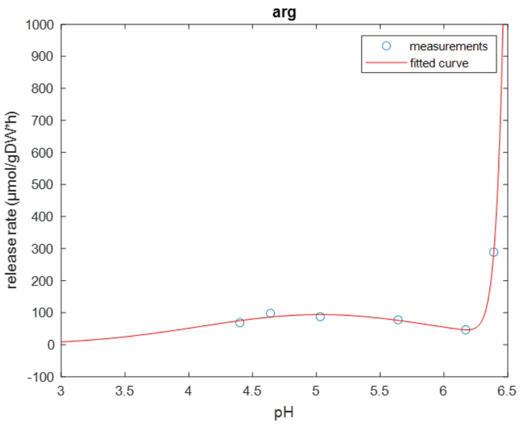

Figure S19. fitted Gauss model for arginine.

General model Gauss2:
val(x) = a1*exp(-((x-b1)/c1)^2) + a2*exp(-((x-b2)/c2)^2)
Coefficients:
a1 =   1.48e+04
b1 =   6.392
c1 =   0.3152
a2 = -1.446e+04
b2 =   6.405
c2 =   0.3381

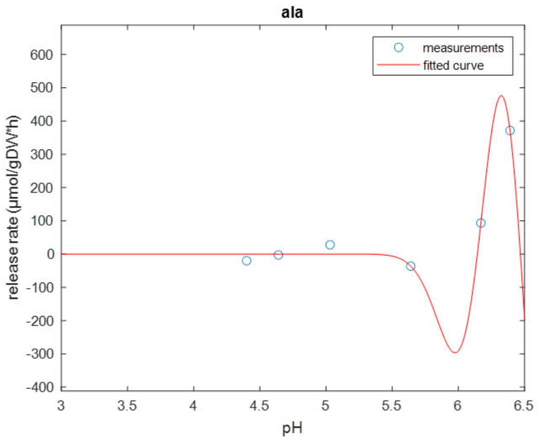

Figure S20. fitted Gauss model for alanine.

General model Gauss2:
val(x) = a1*exp(-((x-b1)/c1)^2) + a2*exp(-((x-b2)/c2)^2)
Coefficients:
a1 =   378.2
b1 =   6.506
c1 =   0.2184
a2 =   76.33
b2 =   5.419
c2 =   0.9316

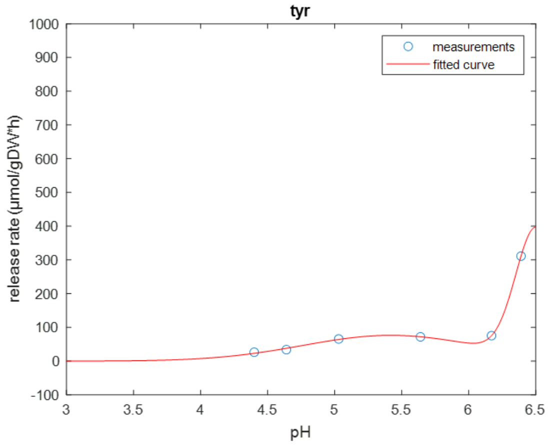

Figure S21. fitted Gauss model for tyrosine.

General model Gauss1:
val(x) = a1*exp(-((x-b1)/c1)^2)
Coefficients (with 95% confidence bounds):
a1 =   520.2 (-268.9, 1309)
b1 =   6.748 (1.102, 12.39)
c1 =   1.785 (-2.652, 6.222)

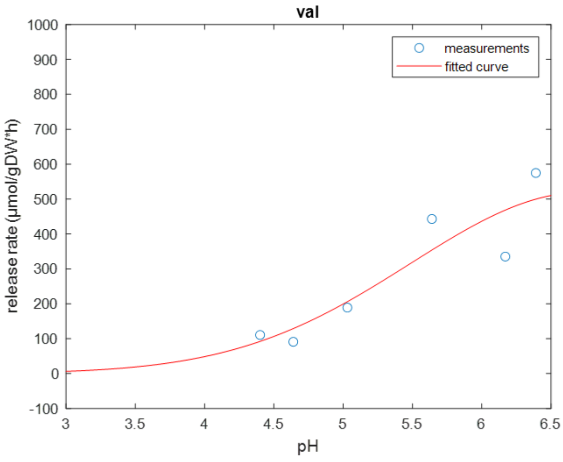

Figure S22. fitted Gauss model for valine.

General model Gauss1:
  val(x) = a1*exp(-((x-b1)/c1)^2)
  Coefficients (with 95% confidence bounds):
    a1 =   76.03 (38.87, 113.2)
    b1 =   5.653 (5.397, 5.91)
    c1 =   0.547 (0.2886, 0.8054)

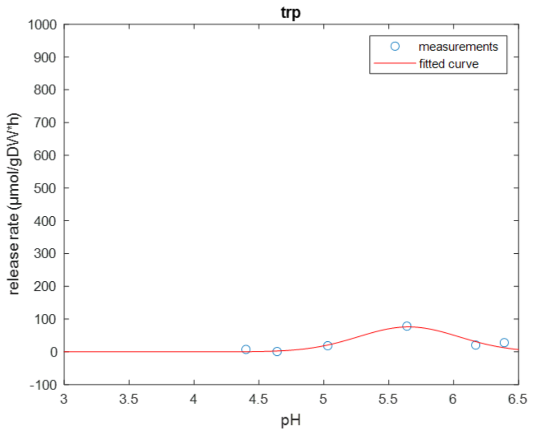

Figure S23. fitted Gauss model for tryptophan.

General model Gauss1:
  val(x) = a1*exp(-((x-b1)/c1)^2)
  Coefficients (with 95% confidence bounds):
    a1 =   150.5 (53.3, 247.7)
    b1 =   5.387 (5.005, 5.77)
    c1 =   0.9245 (0.2981, 1.551)

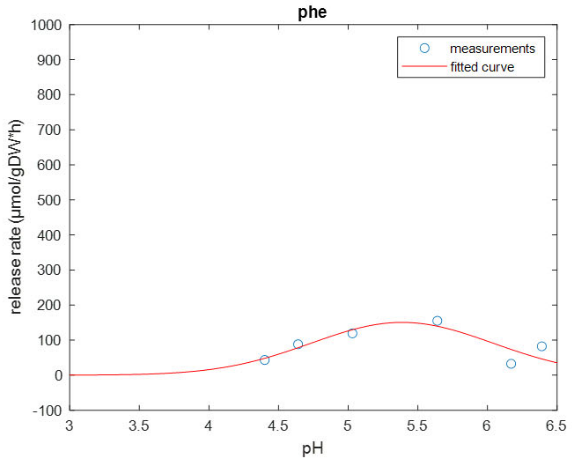

Figure S24. fitted Gauss model for phenylalanine.

General model Gauss2:
  val(x) = a1*exp(-((x-b1)/c1)^2) + a2*exp(-((x-b2)/c2)^2)
  Coefficients:
    a1 =   6.719e+15
    b1 =   9.329
    c1 =   0.5253
    a2 =   117
    b2 =   4.938
    c2 =   1.028

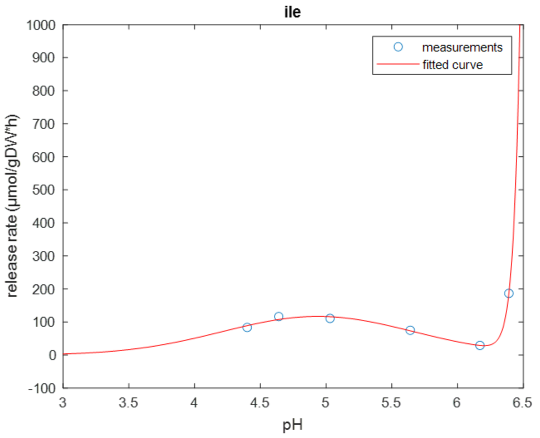

Figure S25. fitted Gauss model for isoleucine.

General model Gauss2:
val(x) = a1*exp(-((x-b1)/c1)^2) + a2*exp(-((x-b2)/c2)^2)
Coefficients:
a1 = 6024
b1 = 5.848
c1 = 0.1445
a2 = 383.4
b2 = 6.15
c2 = 1.985

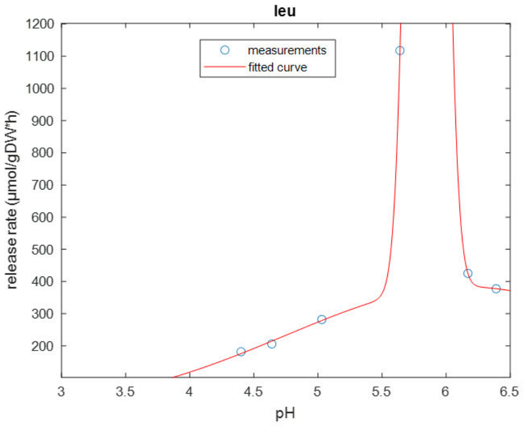

Figure S26. fitted Gauss model for leucine.

General model Gauss2:
val(x) = a1*exp(-((x-b1)/c1)^2) + a2*exp(-((x-b2)/c2)^2)
Coefficients:
a1 = 1.993e+17
b1 = 7.703
c1 = 0.2261
a2 = 124.5
b2 = 5.303
c2 = 0.9719

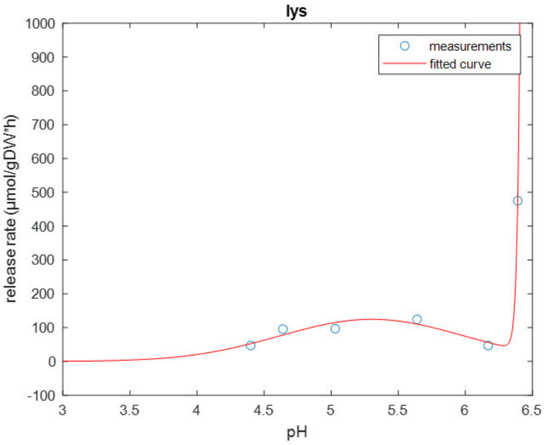

Figure S27. fitted Gauss model for lysine.
